# 2-(Tricyclo[3.3.1.1^3,7^]dec-2-ylamino)ethanol hemihydrate

**DOI:** 10.1107/S1600536808016851

**Published:** 2008-06-07

**Authors:** Grant A. Boyle, Thavendran Govender, Hendrik G. Kruger, Oluseye K. Onajole

**Affiliations:** a School of Chemistry, University of KwaZulu-Natal, Durban, 4000, South Africa; b School of Pharmacy and Pharmacology, University of KwaZulu-Natal, Durban, 4000, South Africa

## Abstract

The title adamantane derivative, C_12_H_21_NO·0.5H_2_O, was synthesized as part of an investigation into the biological activities of cage amino–alcohol compounds as potential anti-tuberculosis agents. The structure displays inter­molecular O—H⋯N, N—H⋯O, O—H⋯O hydrogen bonding and a layered packing structure with distinct hydro­philic and hydro­phobic regions. The water molecule lies on a twofold rotation axis.

## Related literature

For related literature, see: Bogatcheva *et al.* (2006 ▶); du Pont de Nemours and Co. (1969 ▶); Lee *et al.* (2003 ▶); Tripathi *et al.* (2006 ▶). 
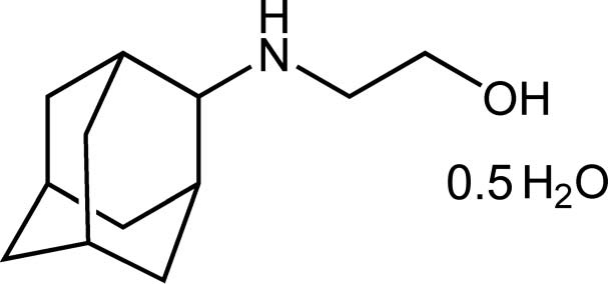


## Experimental

### 

#### Crystal data

C_12_H_21_NO·0.5H_2_O
*M* *_r_* = 204.31
Monoclinic, 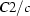

*a* = 11.6739 (3) Å
*b* = 6.5043 (2) Å
*c* = 28.6241 (7) Å
β = 99.8620 (10)°
*V* = 2141.33 (10) Å^3^
*Z* = 8
Mo *K*α radiation
μ = 0.08 mm^−1^
*T* = 173 (2) K
0.56 × 0.43 × 0.18 mm

#### Data collection

Bruker APEXII CCD area-detector diffractometer
Absorption correction: none
13147 measured reflections
2584 independent reflections
2352 reflections with *I* > 2σ(*I*)
*R*_int_ = 0.058

#### Refinement

*R*[*F* ^2^ > 2σ(*F* ^2^)] = 0.042
*wR*(*F* ^2^) = 0.112
*S* = 1.07
2584 reflections
141 parameters
H atoms treated by a mixture of independent and constrained refinement
Δρ_max_ = 0.39 e Å^−3^
Δρ_min_ = −0.19 e Å^−3^

### 

Data collection: *APEX2* (Bruker, 2005 ▶); cell refinement: *SAINT-Plus* (Bruker, 1999 ▶); data reduction: *SAINT-Plus*; program(s) used to solve structure: *SHELXTL* (Sheldrick, 2008 ▶); program(s) used to refine structure: *SHELXTL*; molecular graphics: *Mercury* (Macrae *et al.*, 2006 ▶) and *ORTEP-3* (Farrugia, 1997 ▶); software used to prepare material for publication: *SHELXTL* and *PLATON* (Spek, 2003 ▶).

## Supplementary Material

Crystal structure: contains datablocks I, global. DOI: 10.1107/S1600536808016851/hg2403sup1.cif

Structure factors: contains datablocks I. DOI: 10.1107/S1600536808016851/hg2403Isup2.hkl

Additional supplementary materials:  crystallographic information; 3D view; checkCIF report

## Figures and Tables

**Table 1:** Hydrogen-bond geometry (Å, °)

*D*—H⋯*A*	*D*—H	H⋯*A*	*D*⋯*A*	*D*—H⋯*A*
O1—H1*C*⋯N1^i^	0.84	1.86	2.7007 (12)	175
N1—H1*B*⋯O1*W*^ii^	0.849 (16)	2.398 (16)	3.2241 (12)	164.6 (14)
O1*W*—H1*W*⋯O1^iii^	0.863 (16)	1.963 (17)	2.8147 (12)	168.7 (16)

Symmetry codes: (i) 
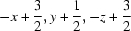
; (ii) 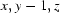; (iii) 
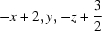
.

**Fig. 1.**
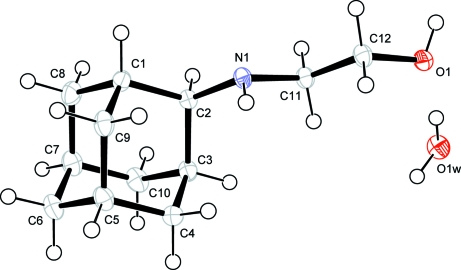
The ORTEP (Farrugia, 1997) diagram of the title compound showing the displacement ellipsoids for non-hydrogen atoms at the 50% probability level.

**Fig. 2.**
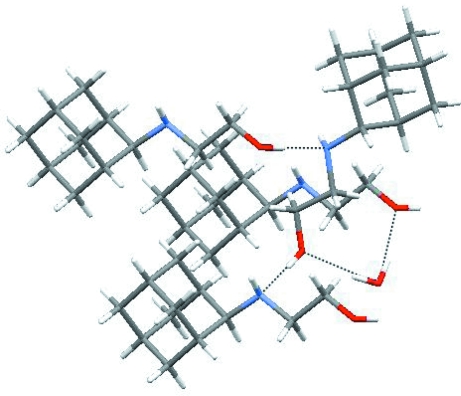
Figure depicting the intermolecular hydrogen bonding.

**Fig. 3.**
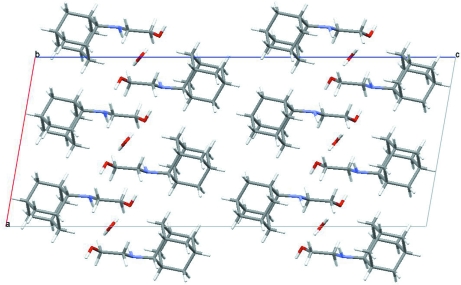
Packing diagram depicting layered structure as seen down the b-axis.

## supplementary crystallographic information

### Comment

The title compound, an adamantane derivative, was synthesized as part of an ongoing study to evaluate the biological activity of such compounds as potential anti-tuberculosis agents (Bogatcheva *et al.* (2006), Lee *et al.* (2003), Tripathi *et al.* (2006)). Although the compound is known (du Pont de Nemours and Co.; 1969), its crystal structure has not been reported.

The compound contains a polycyclic (lipophilic) hydrocarbon region, polar amine and hydroxyl units, and crystallizes with half a water molecule in the asymmetric unit (Fig.1)- the water molecule being situated on a crystallographic 2-fold axis at (1, *y*, 3/4). The title molecule exhibits several C–C bond lengths in the adamantane skeleton that deviate from the expected value of 1.54 Å. This has been observed previously and is typical for these types of compounds.

The structure exhibits intermolecular hydrogen bonding between O1 and N1 of adjacent molecules as well as between O1 and O1W of the water molecule (Fig. 2). There is also a complex network of short contacts between the molecules in structure. These intermolecular interactions result in a layered structure with distinct hydrophilic and hydrophobic regions (Fig. 3). The adamantane skeleton forms the hydrophobic layer while the polar hydroxyl and amino moeties constitute the hydrophilic region.

### Experimental

A mixture of 2-adamantanone (2 g, 13 mmol) and 2-aminoethanol (1 g, 16 mmol) in 20 ml of methanol was stirred under dinitrogen atmosphere at room temperature for 2 h. The mixture was cooled to zero degrees using an external ice bath after with NaBH_4_ (1 g, 26 mmol) was added slowly over a 30 minutes. The mixture was stirred for overnight at room temperature after which it was concentrated *in vacuo *and excess NaBH_4_ was quenched by adding 40 ml of 10% HCl and the product was also extracted as its HCl salt in the process. The aqueous solution was washed with 2x20ml of dichloromethane, after which the aqueous layer was basified (pH 12) with NH_4_OH and the product was extracted from the mixture with dichloromethane (2x30ml), the solvent was dried over Na_2_SO_4_ and concentrated *in vacuo*. The product was recrystallized from dichloromethane, thereby affording pure 2-aminoethanol adamantane (2 g, 77% yield).

### Refinement

Non-hydrogen atoms were first refined isotropically followed by anisotropic refinement by full matrix least-squares calculations based on *F*^2^ using *SHELXTL*. With the exception to H1B and H1W, all hydrogen atoms were first located in the difference map then positioned geometrically, and allowed to ride on their respective parent atoms, with bond lengths of 0.99 Å (CH_2_), 1.00 Å (Methine CH) or 0.84 Å (OH). Isotropic displacement parameters for these atoms were set equal to 1.2 (CH_2_ and CH), or 1.5 OH) times *U_eq_* of the parent atom. Atoms H1B and H1W were located in the difference map and refined freely.

### Crystal data

**Table:**

C_12_H_21_NO·0.5H_2_O	*F*(000) = 904
*M**_r_* = 204.31	*D*_x_ = 1.267 Mg m^−^^3^
Monoclinic, *C*2/*c*	Mo *K*α radiation, λ = 0.71073 Å
Hall symbol: -C 2yc	Cell parameters from 7587 reflections
*a* = 11.6739 (3) Å	θ = 2.9–28.3°
*b* = 6.5043 (2) Å	µ = 0.08 mm^−^^1^
*c* = 28.6241 (7) Å	*T* = 173 K
β = 99.862 (1)°	Plate, colourless
*V* = 2141.33 (10) Å^3^	0.56 × 0.43 × 0.18 mm
*Z* = 8	

### Data collection

**Table:**

Bruker SMART CCD area-detector diffractometer	2352 reflections with *I* > 2σ(*I*)
Radiation source: fine-focus sealed tube	*R*_int_ = 0.058
graphite	θ_max_ = 28.0°, θ_min_ = 1.4°
phi and ω scans	*h* = −15→15
13147 measured reflections	*k* = −8→8
2584 independent reflections	*l* = −37→37

### Refinement

**Table:**

Refinement on *F*^2^	Primary atom site location: structure-invariant direct methods
Least-squares matrix: full	Secondary atom site location: difference Fourier map
*R*[*F*^2^ > 2σ(*F*^2^)] = 0.042	Hydrogen site location: inferred from neighbouring sites
*wR*(*F*^2^) = 0.112	H atoms treated by a mixture of independent and constrained refinement
*S* = 1.07	*w* = 1/[σ^2^(*F*_o_^2^) + (0.0507*P*)^2^ + 1.5961*P*] where *P* = (*F*_o_^2^ + 2*F*_c_^2^)/3
2584 reflections	(Δ/σ)_max_ = 0.001
141 parameters	Δρ_max_ = 0.39 e Å^−^^3^
0 restraints	Δρ_min_ = −0.19 e Å^−^^3^

### Special details

**Table:**

Geometry. All e.s.d.'s (except the e.s.d. in the dihedral angle between two l.s. planes) are estimated using the full covariance matrix. The cell e.s.d.'s are taken into account individually in the estimation of e.s.d.'s in distances, angles and torsion angles; correlations between e.s.d.'s in cell parameters are only used when they are defined by crystal symmetry. An approximate (isotropic) treatment of cell e.s.d.'s is used for estimating e.s.d.'s involving l.s. planes.
Refinement. Refinement of *F*^2^ against ALL reflections. The weighted *R*-factor *wR* and goodness of fit *S* are based on *F*^2^, conventional *R*-factors *R* are based on *F*, with *F* set to zero for negative *F*^2^. The threshold expression of *F*^2^ > σ(*F*^2^) is used only for calculating *R*-factors(gt) *etc*. and is not relevant to the choice of reflections for refinement. *R*-factors based on *F*^2^ are statistically about twice as large as those based on *F*, and *R*- factors based on ALL data will be even larger.

### Fractional atomic coordinates and isotropic or equivalent isotropic displacement parameters (Å^2^)

**Table:**

	*x*	*y*	*z*	*U*_iso_*/*U*_eq_	
C1	0.76341 (9)	−0.06132 (17)	0.61221 (4)	0.0189 (2)	
H1	0.6899	−0.0946	0.6241	0.023*	
C2	0.81420 (9)	0.13986 (16)	0.63482 (3)	0.0156 (2)	
H2	0.7582	0.2520	0.6229	0.019*	
C3	0.92859 (10)	0.18681 (17)	0.61700 (4)	0.0181 (2)	
H3	0.9635	0.3162	0.6321	0.022*	
C4	1.01487 (10)	0.00885 (18)	0.62888 (4)	0.0205 (2)	
H4A	1.0880	0.0411	0.6173	0.025*	
H4B	1.0334	−0.0096	0.6637	0.025*	
C5	0.96251 (10)	−0.18982 (17)	0.60569 (4)	0.0208 (2)	
H5	1.0189	−0.3054	0.6137	0.025*	
C6	0.93562 (11)	−0.16124 (19)	0.55178 (4)	0.0243 (3)	
H6A	1.0082	−0.1301	0.5396	0.029*	
H6B	0.9023	−0.2896	0.5366	0.029*	
C7	0.84905 (11)	0.01531 (19)	0.53962 (4)	0.0240 (3)	
H7	0.8313	0.0338	0.5044	0.029*	
C8	0.73704 (10)	−0.0346 (2)	0.55821 (4)	0.0250 (3)	
H8A	0.7025	−0.1627	0.5433	0.030*	
H8B	0.6802	0.0781	0.5499	0.030*	
C9	0.85024 (10)	−0.23740 (17)	0.62443 (4)	0.0208 (2)	
H9A	0.8159	−0.3668	0.6101	0.025*	
H9B	0.8677	−0.2561	0.6593	0.025*	
C10	0.90159 (11)	0.21388 (18)	0.56282 (4)	0.0241 (3)	
H10A	0.8462	0.3288	0.5546	0.029*	
H10B	0.9739	0.2475	0.5507	0.029*	
C11	0.83975 (9)	0.33596 (16)	0.70953 (4)	0.0170 (2)	
H11A	0.7728	0.4258	0.6973	0.020*	
H11B	0.9109	0.4007	0.7018	0.020*	
C12	0.85067 (10)	0.31373 (16)	0.76292 (4)	0.0186 (2)	
H12A	0.7770	0.2587	0.7706	0.022*	
H12B	0.9132	0.2141	0.7745	0.022*	
N1	0.82297 (8)	0.13298 (14)	0.68680 (3)	0.0156 (2)	
O1	0.87618 (7)	0.50454 (12)	0.78653 (3)	0.01947 (19)	
H1C	0.8167	0.5477	0.7963	0.029*	
O1W	1.0000	0.82045 (19)	0.7500	0.0240 (3)	
H1B	0.8774 (13)	0.053 (2)	0.6990 (5)	0.024 (4)*	
H1W	1.0445 (14)	0.737 (3)	0.7380 (6)	0.037 (4)*	

### Atomic displacement parameters (Å^2^)

**Table:**

	*U*^11^	*U*^22^	*U*^33^	*U*^12^	*U*^13^	*U*^23^
C1	0.0192 (5)	0.0197 (5)	0.0178 (5)	−0.0041 (4)	0.0030 (4)	−0.0029 (4)
C2	0.0175 (5)	0.0146 (5)	0.0146 (5)	0.0007 (4)	0.0027 (4)	0.0003 (4)
C3	0.0224 (5)	0.0155 (5)	0.0172 (5)	−0.0032 (4)	0.0059 (4)	−0.0004 (4)
C4	0.0178 (5)	0.0236 (6)	0.0200 (5)	0.0000 (4)	0.0033 (4)	−0.0024 (4)
C5	0.0247 (6)	0.0180 (5)	0.0198 (5)	0.0045 (4)	0.0038 (4)	−0.0010 (4)
C6	0.0310 (6)	0.0236 (6)	0.0193 (5)	0.0008 (5)	0.0072 (4)	−0.0046 (4)
C7	0.0313 (6)	0.0265 (6)	0.0138 (5)	0.0015 (5)	0.0030 (4)	0.0010 (4)
C8	0.0245 (6)	0.0296 (6)	0.0188 (5)	−0.0003 (5)	−0.0023 (4)	−0.0036 (4)
C9	0.0300 (6)	0.0137 (5)	0.0189 (5)	−0.0029 (4)	0.0048 (4)	−0.0006 (4)
C10	0.0339 (6)	0.0206 (6)	0.0195 (5)	0.0004 (5)	0.0092 (4)	0.0040 (4)
C11	0.0207 (5)	0.0133 (5)	0.0170 (5)	0.0003 (4)	0.0033 (4)	−0.0009 (4)
C12	0.0239 (5)	0.0151 (5)	0.0172 (5)	−0.0006 (4)	0.0049 (4)	−0.0010 (4)
N1	0.0189 (4)	0.0131 (4)	0.0149 (4)	0.0008 (3)	0.0033 (3)	−0.0003 (3)
O1	0.0195 (4)	0.0188 (4)	0.0208 (4)	−0.0012 (3)	0.0054 (3)	−0.0058 (3)
O1W	0.0293 (6)	0.0175 (6)	0.0259 (6)	0.000	0.0064 (5)	0.000

### Geometric parameters (Å, °)

**Table:**

C1—C9	1.5297 (16)	C7—C8	1.5287 (17)
C1—C8	1.5334 (15)	C7—C10	1.5316 (17)
C1—C2	1.5334 (14)	C7—H7	1.0000
C1—H1	1.0000	C8—H8A	0.9900
C2—N1	1.4745 (12)	C8—H8B	0.9900
C2—C3	1.5395 (15)	C9—H9A	0.9900
C2—H2	1.0000	C9—H9B	0.9900
C3—C4	1.5339 (15)	C10—H10A	0.9900
C3—C10	1.5388 (15)	C10—H10B	0.9900
C3—H3	1.0000	C11—N1	1.4700 (13)
C4—C5	1.5314 (16)	C11—C12	1.5187 (14)
C4—H4A	0.9900	C11—H11A	0.9900
C4—H4B	0.9900	C11—H11B	0.9900
C5—C9	1.5305 (16)	C12—O1	1.4200 (13)
C5—C6	1.5324 (15)	C12—H12A	0.9900
C5—H5	1.0000	C12—H12B	0.9900
C6—C7	1.5299 (17)	N1—H1B	0.849 (16)
C6—H6A	0.9900	O1—H1C	0.8400
C6—H6B	0.9900	O1W—H1W	0.863 (16)
			
C9—C1—C8	109.03 (9)	C6—C7—C10	109.54 (10)
C9—C1—C2	110.44 (9)	C8—C7—H7	109.5
C8—C1—C2	108.97 (9)	C6—C7—H7	109.5
C9—C1—H1	109.5	C10—C7—H7	109.5
C8—C1—H1	109.5	C7—C8—C1	109.82 (9)
C2—C1—H1	109.5	C7—C8—H8A	109.7
N1—C2—C1	110.79 (8)	C1—C8—H8A	109.7
N1—C2—C3	115.22 (8)	C7—C8—H8B	109.7
C1—C2—C3	108.91 (8)	C1—C8—H8B	109.7
N1—C2—H2	107.2	H8A—C8—H8B	108.2
C1—C2—H2	107.2	C1—C9—C5	110.01 (9)
C3—C2—H2	107.2	C1—C9—H9A	109.7
C4—C3—C10	108.86 (9)	C5—C9—H9A	109.7
C4—C3—C2	110.53 (9)	C1—C9—H9B	109.7
C10—C3—C2	108.48 (9)	C5—C9—H9B	109.7
C4—C3—H3	109.6	H9A—C9—H9B	108.2
C10—C3—H3	109.6	C7—C10—C3	109.77 (9)
C2—C3—H3	109.6	C7—C10—H10A	109.7
C5—C4—C3	110.03 (9)	C3—C10—H10A	109.7
C5—C4—H4A	109.7	C7—C10—H10B	109.7
C3—C4—H4A	109.7	C3—C10—H10B	109.7
C5—C4—H4B	109.7	H10A—C10—H10B	108.2
C3—C4—H4B	109.7	N1—C11—C12	109.98 (8)
H4A—C4—H4B	108.2	N1—C11—H11A	109.7
C9—C5—C4	108.72 (9)	C12—C11—H11A	109.7
C9—C5—C6	109.72 (9)	N1—C11—H11B	109.7
C4—C5—C6	109.38 (9)	C12—C11—H11B	109.7
C9—C5—H5	109.7	H11A—C11—H11B	108.2
C4—C5—H5	109.7	O1—C12—C11	111.73 (8)
C6—C5—H5	109.7	O1—C12—H12A	109.3
C7—C6—C5	109.49 (9)	C11—C12—H12A	109.3
C7—C6—H6A	109.8	O1—C12—H12B	109.3
C5—C6—H6A	109.8	C11—C12—H12B	109.3
C7—C6—H6B	109.8	H12A—C12—H12B	107.9
C5—C6—H6B	109.8	C11—N1—C2	113.60 (8)
H6A—C6—H6B	108.2	C11—N1—H1B	109.6 (10)
C8—C7—C6	109.42 (10)	C2—N1—H1B	110.6 (10)
C8—C7—C10	109.34 (10)	C12—O1—H1C	109.5
			
C9—C1—C2—N1	−69.54 (11)	C6—C7—C8—C1	60.43 (12)
C8—C1—C2—N1	170.72 (9)	C10—C7—C8—C1	−59.53 (12)
C9—C1—C2—C3	58.18 (11)	C9—C1—C8—C7	−60.00 (12)
C8—C1—C2—C3	−61.56 (11)	C2—C1—C8—C7	60.61 (12)
N1—C2—C3—C4	67.40 (11)	C8—C1—C9—C5	59.43 (11)
C1—C2—C3—C4	−57.78 (11)	C2—C1—C9—C5	−60.27 (11)
N1—C2—C3—C10	−173.32 (9)	C4—C5—C9—C1	60.16 (11)
C1—C2—C3—C10	61.50 (11)	C6—C5—C9—C1	−59.42 (11)
C10—C3—C4—C5	−59.70 (12)	C8—C7—C10—C3	59.75 (12)
C2—C3—C4—C5	59.36 (11)	C6—C7—C10—C3	−60.14 (12)
C3—C4—C5—C9	−59.71 (11)	C4—C3—C10—C7	59.61 (12)
C3—C4—C5—C6	60.09 (12)	C2—C3—C10—C7	−60.72 (12)
C9—C5—C6—C7	59.30 (12)	N1—C11—C12—O1	175.36 (9)
C4—C5—C6—C7	−59.87 (12)	C12—C11—N1—C2	−178.67 (8)
C5—C6—C7—C8	−59.80 (12)	C1—C2—N1—C11	−164.04 (9)
C5—C6—C7—C10	60.05 (12)	C3—C2—N1—C11	71.76 (11)

### Hydrogen-bond geometry (Å, °)

**Table:**

*D*—H···*A*	*D*—H	H···*A*	*D*···*A*	*D*—H···*A*
O1—H1C···N1^i^	0.84	1.86	2.7007 (12)	175
N1—H1B···O1W^ii^	0.849 (16)	2.398 (16)	3.2241 (12)	164.6 (14)
O1W—H1W···O1^iii^	0.863 (16)	1.963 (17)	2.8147 (12)	168.7 (16)

Symmetry codes: (i) −*x*+3/2, *y*+1/2, −*z*+3/2; (ii) *x*, *y*−1, *z*; (iii) −*x*+2, *y*, −*z*+3/2.
